# Earache

**Published:** 1886-10

**Authors:** W. Cheatham

**Affiliations:** Louisville, Ky.; Lecturer on Diseases of Eye, Ear, Throat and Nose, University of Louisville; Eye, Ear, Throat and Nose Physician, Louisville City Hospital; Masonic Widows and Orphans’ Home of Kentucky, Etc.


### EARACHE.

BY W. CHEATHAM, M. D., LECTURER ON DISEASES OF EYE, EAR, THROAT AND NOSE, UNIVERSITY OF LOUISVILLE; EYE, EAR, THROAT AND NOSE PHYSICIAN, LOUISVILLE CITY HOSPITAL; MASONIC WIDOWS AND ORPHANS' HOME OF KENTUCKY, ETC.

Earache, or acute catarrh of middle ear, is, when taken in time, one of the easiest of the affections of this organ to manage; from mistreated and neglected cases come most of the hopeless cases of non-suppurative and suppurative inflammations of the middle ear. This may originate as an inflammation of the middle ear itself, or as an extension from an inflammation of throat and eustachian tube, or an inflammation of external ear. Whatever its origin, the indications for local treatment are the same. Its management should be a little different from that of inflammation of other parts of the body symptoms. It would be a little difficult to mistake a well marked case of earache; in small children and infants, such a mistake is possible. I have seen otalgia or neuralgia of ear, and inflammation of external auditory canal, mistaken for earache or acute catarrh of middle ear. Either can be easily diagnosed if the proper instruments for examination are at hand. When not, it is somewhat more difficult. Firm pressure on tragus or manipulation of auricle may locate the trouble in ear, and so diagnose it from a case of otalgia. With an infant a diagnosis may be correctly made by this maneuver. The symptoms of earache and some of the inter-cranial troubles in children are quite similar. A mistake in such cases might prove quite serious.

In earache there is most always an elevation of temperature, slight in some, and again in others quite high. There is deep-seated pain, varying much in different cases, in some slight, and in others as severe as any pain ever suffered. I have seen many a strong man roll and tumble, declaring no suffering could be as acute. This pain is usually increased by pressure over external auditory canal. There are often noises in the ear and usually deafness. In some cases, though, the ear is hyperæsthetic, and external noises are extremely annoying. On looking into an ear in which we find acute catarrh of middle ear, we may see anything from a slight degree of hyperæmia to that of an intensely inflammed drum-head, bulging and obliterating all landmarks. Often, if in the first stages, there can nothing abnormal be seen but a slight increase of the number of blood-vessels around the rim of the drum membrane, with possibly a few vessels running along the handle of malleus. Again, there is simply a diffuse redness of membrana tympani. Again, where there is fluid in tympanic cavity, the drum-head will not only be inflamed greatly, but pushed out, or bulge into the external auditory canal. With this we may have had a rigor, or rigors, with an inflamed throat, the pain extending up the course of the eustachian tube to middle ear.

The management of this inflammation differs in its different degrees. At the head of the list of remedies in this affection I place blood-letting; this is indicated in all acute stages. Leeches are the best means. Two, three or four leeches (depending upon the condition of the patient as to how much blood he can spare, etc.) in the tragus, some of them well in on anterior wall of external auditory canal (first plugging canal with common, not absorbent cotton), allowing them to remain until they drop off themselves, and permit the bites to bleed for half an hour afterwards. This line of treatment is intended for those cases in which there is no fluid in middle ear. If this does not relieve the pain an opiate may be given. Opium should be used cautiously in earache, for if there be fluid in the middle ear, it should be evacuated, and one of the symptoms of its presence, pain, may be concealed by an opiate. Therein lies the danger of narcotics in the treatment of acute catarrh of middle ear. Next to blood-letting, I place hot water, flowing into ear in a steady stream from either a douche fountain syringe, or if nothing else can be gotten, to place a basin of water higher than the head, and start a siphon by means of a piece of rubber tubing or a Davidson's syringe. The ear should be pulled well up and back, the small tip pushed well into canal, and the syringe or basin not too high, or else it will produce vertigo and pain; the water should be started warm and made warmer gradually until it is as hot as can be borne. The ear should then be thoroughly dried by means of absorbent cotton brushes, made by twisting a little of the cotton on a match or wooden tooth pick, leaving the end well protected, and afterwards the ear packed with warm cotton and over this a warm flannel or a salt or hops compress. This should be repeated every one, two, three or four hours as indicated. It is well to do this in some cases, even after the ear has been leeched. If it is impossible to get any kind of a syringe, warm water can be put in the ear with a spoon or hot air substituted.

A word as to the hot poultices, which is the most common remedy for earache with the laity and the mass of the medical profession. There is no doubt that many cases of acute catarrh of the middle ear go on to resolution, and leave the ear in a good condition under the use of the poultice. But again, the majority of such cases go on to suppuration, perforation of drum membrane with all its bad effects. By the use of the hot water douche, we get all the good effects of the poultice with none of its bad. The use of the hot water is fully as simple and efficacious, and many times less dangerous. The poultice either relieves by resolution (which it does in about one-third of the cases) or by suppuration with perforation of drum membrane in the other two-thirds of the cases. Before the drum membrane perforates, it has become very much softer and macerated by the continued poulticing; and when the break occurs, a greater part of it is swept away, and we have, as a result, chronic suppuration of middle ear with all its dangers—polypi, necrosis of bone, abscess of brain, or meningitis and death.

Again, when poultices are used, eczema and furuncles of external auditory canal, with frequent relapses, often follow. What a different history when heated as I direct. Drops of olive oil, laudanum, glycerine, cocaine muriate, atrop. sulph., morphia, etc., are usually of little service in a genuine case of acute catarrh of middle ear. If put in warm, the heat of course does good. After the leeches, hot water and morphia are used; suppose the disease progresses, notwithstanding, and we find now the drum membrane bulging, and other indications that there is pent-up fluid in the middle ear cavity, what are the indications ? Here, as elsewhere, that is evacuated. A needle or a pin with a stick for a holder, a knitting-needle with a sharpened point, a regular paracentesis needle or whatever may be on hand, use it.

Some time ago I was called to see a lady suffering intensely with earache. Found bulging drum, etc. Had no instrument with me with which to perform paracentesis. I found an old crochet needle that could be bent. I tied to this a common sewing needle. With this I punctured the drum membrane, and gave almost instant relief. The puncture should be either made low down or where the membrane bulges the most. This in some cases has to be repeated several times. Again, in some cases, it is better to make an incision. After this, Valsalva should be performed, and all fluid in the cavity blown out. Leeches if indicated should be used, and hot water douche. Usually, or in a great majority of these cases, the puncture or incision made in the drum membrane heals too rapidly, and the operation has to be repeated. Instead of a hole in the drum membrane that will never heal, and as a result chronic suppuration of middle ear with all its evils, we have, by the treatment just laid out, a solid membrane and a good ear. The more modern treatment is no more difficult to carry out than the old, and the results are so different that comparison is out of the question.

As I have stated before, if the aurist could get hold of all the affections of the middle ear, and especially of all cases of acute catarrh of the middle ear in its first stages, there would be but little necessity of a chapter being written upon chronic suppurative and non-suppurative inflammations of the middle ear, and we would have but few cases of middle ear deafness, mastoid cell disease, cerebral abscess, meningitis, etc. Of course during this time the general health should be attended to, and the treatment directed to any affection of the throat and eustachian tube. Many of these cases in this section of the country are malarial, and are relieved by calomel and quinine. In many of the cases of simple earache the Politzer bags will always give relief. Inflate the ear with it, or by some other means, and relief will usually be immediate. To prevent relapses, the general treatment, constitutional and hygienic, and the local to throat, with inflation of middle ear, with or without catheter, should be continued for some time after all acute symptoms have disappeared. Inflation keeps the eustachian tube open, forces air into middle ear, and by keeping up motion of drum membrane prevents anchylosis of the small bones of that cavity.

